# Identification of Fouling Occurring during Coupled Electrodialysis and Bipolar Membrane Electrodialysis Treatment for Tofu Whey Protein Recovery

**DOI:** 10.3390/membranes14040088

**Published:** 2024-04-11

**Authors:** Rosie Deschênes Gagnon, Marie-Ève Langevin, Florence Lutin, Laurent Bazinet

**Affiliations:** 1 Institute of Nutrition and Functional Foods (INAF), Food Science Department, Laboratoire de Transformation Alimentaire et Procédés ÉlectroMembranaires (LTAPEM/Laboratory of Food Processing and ElectroMembrane Processes), Université Laval, Quebec City, QC G1V 0A6, Canada; rosie.deschenes-gagnon.1@ulaval.ca; 2 Eurodia Industrie S.A.S—Zac Saint Martin, Impasse Saint Martin, 84120 Pertuis, France; melangevin@ameridia.com (M.-È.L.); florence.lutin@eurodia.com (F.L.)

**Keywords:** fouling, scaling, electrodialysis, electro-acidification, bipolar membrane, isoflavones, tofu whey

## Abstract

Tofu whey, a by-product of tofu production, is rich in nutrients such as proteins, minerals, fats, sugars and polyphenols. In a previous work, protein recovery from tofu whey was studied by using a coupled environmental process of ED + EDBM to valorize this by-product. This process allowed protein recovery by reducing the ionic strength of tofu whey during the ED process and acidifying the proteins to their isoelectric point during EDBM. However, membrane fouling was not investigated. The current study focuses on the fouling of membranes at each step of this ED and EDBM process. Despite a reduction in the membrane conductivities and some changes in the mineral composition of the membranes, no scaling was evident after three runs of the process with the same membranes. However, it appeared that the main fouling was due to the presence of isoflavones, the main polyphenols in tofu whey. Indeed, a higher concentration was observed on the AEMs, giving them a yellow coloration, while small amounts were found in the CEMs, and there were no traces on the BPMs. The glycosylated forms of isoflavones were present in higher concentrations than the aglycone forms, probably due to their high amounts of hydroxyl groups, which can interact with the membrane matrices. In addition, the higher concentration of isoflavones on the AEMs seems to be due to a combination of electrostatic interactions, hydrogen bonding, and π–π stacking, whereas only π–π stacking and hydrogen bonds were possible with the CEMs. To the best of our knowledge, this is the first study to investigate the potential fouling of BPMs by polyphenols, report the fouling of IEMs by isoflavones and propose potential interactions.

## 1. Introduction

Tofu whey is a by-product of tofu production, which is generated in very large quantities. Every kilogram of tofu produces 9 kg of tofu whey. With the annual growth rate of tofu being approximately 5.2% from 2019 to 2025, the quantity of tofu whey constantly increases every year [1]. This by-product is a complex solution composed of proteins, minerals, sugars, fats and polyphenols. No recovery method is currently incorporated into tofu production, and tofu whey is considered a waste product. To valorize this waste, a combined process, coupling electrodialysis and bipolar membrane electrodialysis (ED + EDBM), both technologies recognized as green, has previously been studied to recover proteins from tofu whey. During the ED phase, the tofu whey was demineralized, and therefore, the ionic strength was lowered. This allowed protein precipitation during the second phase, consisting of the electro-acidification of tofu whey by EDBM [2,3]. In their previous work on protein recovery from tofu whey by electrodialytic processes, Deschênes Gagnon et al. (2023) reported a pH variation in the tofu whey and recovery solution during the ED phase, suggesting the occurrence of water splitting [3]. It is well known that water splitting increases the scaling propensity of IEMs [4]. In addition, an increase in the global system resistance was reported during the EDBM phase, which indicated a potential change in the membrane integrity or potential membrane fouling [4,5]. However, this study focused on the technological feasibility of the coupled process as well as the identification of precipitated proteins, and not on the investigation of membrane fouling.

Membrane fouling is an important limitation often encountered during electrodialysis processes. Consequently, identifying the molecule(s) responsible for fouling as well as their fouling mechanism(s) is the main concern to limit the negative effects of fouling. Indeed, fouling can be reversible, referring to the adsorption or deposition of undesirable molecules on the surface of a membrane., which can be removed by physical cleaning [6,7]. Irreversible fouling refers to the adsorption of molecules inside the membrane due to different interactions, where they can only be removed by chemical cleaning [6,7]. In addition, there are also different types of fouling: colloidal, organic, mineral and biological, which can be caused by molecules of various natures, such as proteins, minerals, polysaccharides and polyphenols [6,8,9,10,11,12]. Membrane fouling affects the performance and the cost of ED processes by increasing the electrical resistance and decreasing the permselectivity of the membranes, leading to membrane integrity alteration [13,14,15]. The fouling of ion-exchange membranes has been studied multiple times, identifying the species causing fouling and their interactions with the membranes. Many studies have been conducted on the fouling by minerals (specifically named scaling), proteins and polyphenols such as anthocyanins and tannins [16,17,18,19], but no information is available on the nature of the potential fouling by tofu whey components.

The aim of this study was to investigate the fouling of membranes at each step of the coupled ED + EDBM process for the extraction of protein from tofu whey. The objectives were to (1) characterize the membranes before and after each step of the ED + EDBM treatments, (2) identify the nature of the membrane fouling, (3) identify the species interacting with each type of membrane used in this coupled process, and (4) propose the possible interactions and a tentative explanation of the fouling mechanisms.

## 2. Materials and Methods

### 2.1. Materials

The tofu whey was provided by Unisoya (Saint-Isidore-de-Laprairie, QC, Canada). The proximal composition of the tofu whey is presented in Table 1. The membranes used were Neosepta CMX-fg, AMX-fg and BP-1E from Astom (Tokyo, Japan). The structural characteristics of the membranes are presented in Table 2.

### 2.2. Protocol

**Figure 1:**
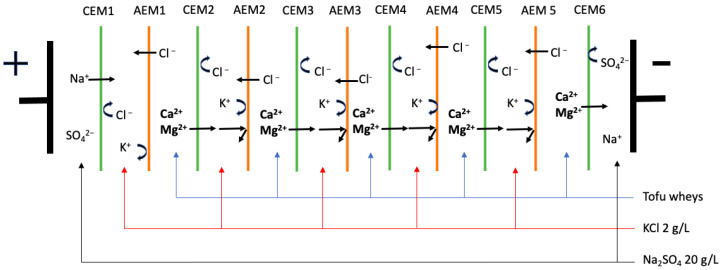
Configuration of ED cell used for demineralization of tofu whey.

**Figure 2:**
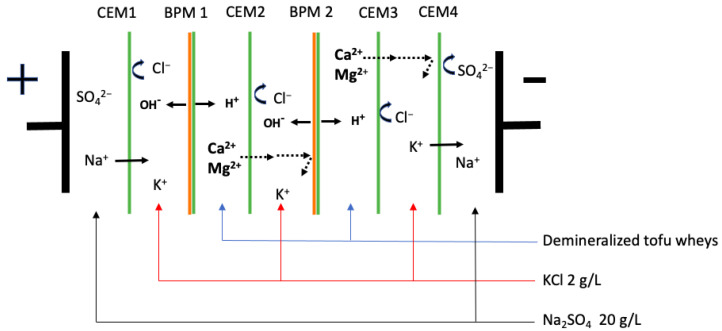
Configuration of EDBM cell used for acidification of demineralized tofu whey.

As described by Deschênes Gagnon et al. ([3], the tofu whey was pre-concentrated at 3 volume concentration factors (VCF) (1×, 2× and 3×) by nanofiltration (NF). Then, the successive ED and EDBM processes were performed using an MP-type cell (ElectroCell AB, Täby, Sweden) with an effective surface of 100 cm^2^ [3]. For the ED process, the tofu wheys were demineralized until 70% demineralization was achieved. The configuration consisted of six cation-exchange membranes (CEMs) and five anion-exchange membranes (AEMs) (Figure 1). Then, the demineralized tofu wheys were collected for electro-acidification by EDBM. The cell configuration consisted of two bipolar membranes (BPMs) and four cation-exchange membranes (Figure 2). EDBM was performed until the tofu wheys reached pH 4.4. For both electrodialytic processes, the tofu whey, recovery solution (700 mL, 2 g/L KCl), and electrode rinsing solution (800 mL, 20 g/L Na_2_SO_4_) circulated between the membranes at flow rates of 700, 700 and 1000 mL/min, respectively. The voltage was maintained at 9 V throughout both processes.

For each tofu whey concentration, to ensure the presence or absence of fouling, as reported by Casademont et al. (2008), three consecutive treatments were carried out by replacing all the solutions with fresh ones but leaving the membranes in place and running until the end of the ED or EDBM process [26]. After the ED + EDBM treatments, a water rinse was performed and the membranes were removed from the MP cell. For the purpose of this work and further analyses, only the membranes used to treat the 3X concentrated tofu whey were used, as these were the most susceptible to fouling. From the ED process, AEM2, AEM3, CEM3 and CEM4 were selected, while from the EDBM process, BPM1, BPM2, CEM2 and CEM3 were selected, as these were the most representative of the processes and the most susceptible to fouling due to their direct contact with the tofu whey. They were compared to pristine membranes.

### 2.3. Membrane Characterization

#### 2.3.1. Membrane Thickness and Electrical Conductivity

The membrane thickness and electrical conductivity were measured before and after 3 runs, as described by Lemay et al. (2019) [27]. Prior to the analysis, the membranes were soaked in 0.5 M NaCl solution for 30 min. 

For the thickness measurement, an electronic digital device (Marathon Watch Company LTD., Richmond Hill, ON, Canada) was used. Six measurements were taken at different locations on the membrane, and the average thickness was calculated.

The membrane electrical conductivity was calculated using the membrane thickness measurements and the electrical resistance obtained from the membrane conductance (G). The membrane conductance was measured using a YSI conductivity meter model 3100 (Yellow Springs Instrument Co., Yellow Springs, OH, USA) equipped with a specially designed clip from the Laboratoire des Matériaux Échangeurs d’Ions (Université Paris XII, Créteil, Val de Marne, France). Six measurements were taken at different locations on the membranes, and the average conductance was used to calculate the conductivity [27].

The membrane electrical resistance was calculated according to Lteif et al. (1999) and Lebrun et al. (2003) [28,29], using Equation (1):

(1) $$R_{m}{=\frac{1}{G_{m}}=\frac{1}{G_{m+s}}-\frac{1}{G_{s}}=R}_{m+s}-R_{s}$$

where Rm is the transverse electric resistance of the membrane (in Ω), R_m+s_ is the resistance of the membrane and reference solution measured together (in Ω), and Rs is the resistance of the reference solution (in Ω).

The membrane electrical conductivity κ (S/cm) was calculated according to Lteif et al. (1999) [28], using Equation (2):

(2) $$\kappa =\frac{L}{R_{m}A}$$

where L is the membrane thickness (in cm) and A is the electrode area (1 cm^2^).

#### 2.3.2. Ash and Mineral Contents

The ash content of the membranes was determined according to the AOAC method 945.46 [30]. First, membrane coupons of 4 cm^2^ were weighted into pre-weighted crucibles and placed in a furnace (Lindberg/Blue M Moldatherm Box Furnaces, Thermo Fisher Scientific, Waltham, MA, USA) at 550 °C for 24 h and weighed [31]. The analysis was performed in triplicate, and the ash content was calculated using Equation (3):

(3) $$\mathrm{Ash}\mathrm{content}=\left(\frac{\mathrm{Sample}\mathrm{mass}\mathrm{after}\mathrm{incineration}}{\mathrm{Sample}\mathrm{mass}\mathrm{before}\mathrm{incineration}}\right)\times 100$$

To determine the mineral content, the method described by Dufton et al. (2018) was used [4]. Ash samples were solubilized in 2 mL of 25% nitric acid and 8 mL of miliQ water. The solutions were filtered with a 0.45 µm PTFE filter (CHROMESPEC Syring Filter, Chromatographic Specialties, Brockville, ON, Canada). Calcium, magnesium, potassium, sodium, and phosphorus were determined using an Agilent 5110 SVDV ICP-OES (Agilent Technologies, VC, Australia), using the following wavelengths: 393.366, 396.847, 422.673 (Ca); 766.491 (K); 279.553, 280.270, 285.213 (Mg); 588.995, 589.592 (Na); 177.434, 178.222, 213.618, 214.914 (P). The analyses for all the ions were carried out in radial and/or axial view. The analysis was carried out in triplicate, and the results were expressed in g/100 g of membrane on a dry basis. The limit of detection was parts per billion (ppb) [32].

#### 2.3.3. Ion-Exchange Capacity

The ion-exchange capacity (IEC) corresponding to the number of active sites was measured by soaking CEM coupons of 4 cm^2^ in 1 N HCl overnight and rinsing with demineralized water. Then, the membranes were soaked again in a known volume of 0.1 N NaOH for 15 min. The membranes were then rinsed with distilled water, and the rinsed water was mixed with the NaOH solution and titrated with 0.5 N HCl. The analysis was performed in triplicate and the IEC was calculated according to Equation (4), expressed in meq per gram of dry membrane.

(4) $$IEC=\frac{\left(VNaOH\times \left[NaOH\right]\right)-\left(VHCl\times \left[HCl\right]\right)}{Mm}$$

where IEC is the number of milliequivalents per gram of dry membrane, VNaOH and [NaOH] are the volume (mL) and the concentration (N) of NaOH solution, VHCl and [HCl] are the volume (mL) and the concentration (N) of the HCl solution used for the titration of the NaOH solution and Mm is the mass of the dry membrane (g).

To measure the IEC of the AEMs, AEM coupons of 4 cm^2^ were converted to a chloride form by overnight immersion in 1 N HCl solution. Subsequently, excessive Cl- ions were removed from the membrane after rinsing them with demineralized water. The membranes were then immersed in 50 mL of Na_2_SO_4_ (0.25 N) solution for one hour, and the released chloride ions were titrated with a 0.05 N AgNO_3_ solution with K_2_CrO_4_ as an indicator (Mohr method). The analysis was carried out in triplicate and the IEC value of the AEMs, expressed in meq per gram of dry membrane, was calculated using Equation (5):

(5) $$\mathrm{IEC}=\frac{\left[{\mathrm{AgNO}}_{3}\right]\times V_{{\mathrm{AgNO}}_{3}}}{\mathrm{Mm}}$$

where IEC is the number of milliequivalents per gram of dry membrane, V_AgNO_3__ and [AgNO_3_] are the volume (mL) and the concentration (N) of the AgNO_3_ solution used for the titration of the Na_2_SO_4_ solution, and Mm is the mass of the dry membrane (g).

#### 2.3.4. Scanning Electron Microscopy and X-ray Elemental Analysis (SEM/EDX)

Images of both sides of the pristine and used membranes were taken using a scanning electron microscope (SEM) (Quanta 3D FEG, Hillsboro, OR, USA). The conditions were 5 kV accelerating voltage with a 9.5–10.5 mm working distance and 400× magnification. The microscope was equipped with an energy dispersive spectrometer (EDX) detector to perform elemental analyses (PV8206/60 Genesis XM2, EDAX, Tokyo, Japan) [33]. The analysis was carried out in triplicate.

#### 2.3.5. ATR-FTIR Analysis

Both sides of the pristine and used membranes were analyzed by attenuated total reflection–Fourier transform infrared (ATR–FTIR) spectroscopy (Nicolet™ 6700, Thermo Fisher Scientific, Madison, WI, USA). A spectral resolution of 4 cm^−1^ was used, with 128 scans for each spectrum. All the samples were analyzed in absorption mode using an ATR element with a diamond crystal [34]. The analysis was carried out in triplicate.

#### 2.3.6. Isoflavone Contents

For the isoflavone extraction, the method described by Bdiri et al. (2020) for anthocyanin extraction [17] was used. Briefly, 9 cm^2^ membrane coupons were weighted and ground using a CryoMill (25 Hz, 2 min, CryoMill, Retsch, Germany). Then, in 1.5 mL Eppendorf tubes, ground membranes were soaked in 4% m/V of a mix of a solvent composed of 25% acetonitrile, 25% methanol, 25% isopropanol, 25% miliQ water for 24 h under agitation. The solutions were then centrifuged at 5000 rpm for 5 min at room temperature to remove the membrane residues, and the supernatant was collected. The solutions were vacuum dried (Savant SPD131DDA SpeedVac concentrator, Thermo Scientific, MS, Waltham, MA, USA). The samples were reconstituted in 300 µL of MeOH 80% at a 5× concentration, solubilized for 10 min with ultrasound and filtered through a 0.22 µm nylon filter. Then, the samples were analyzed by reversed-phase HPLC using a Waters Acquity UPLC coupled to a PDA detector and a Waters triple quadrupole mass spectrometer (TQD) equipped with an electrospray source (Milford, MA, USA). Next, 1 µL of sample was injected on a HSS T3 Premier column (2.1 mm × 100 mm, 1.8 µm) to separate the isoflavones. The mobile phase consisted of 0.1% acetic acid (solvent A) and acetonitrile (solvent B), the flow rate was 0.5 mL/min and gradient elution was as follows: 0–0.36 min, 10% B; 0.36–1 min, 10–22% B; 1–2.4 min, 22% B; 2.4–4.5 min, 22–90% B; 4.5–5 min, 90% B; 5–8 min, 10% B. Chromatographic data were acquired at 260 nm. Standards of isoflavones were used to compare the retention time. Mass spectrometry was used to confirm the identification in multiple reaction monitoring mode using parameters described by Zhang et al. (2017) [35]. The analysis was carried out in triplicate and the results expressed in mg of isoflavones per g of dry membrane.

## 3. Results

### 3.1. Membrane Thickness and Conductivity

**Figure 3:**
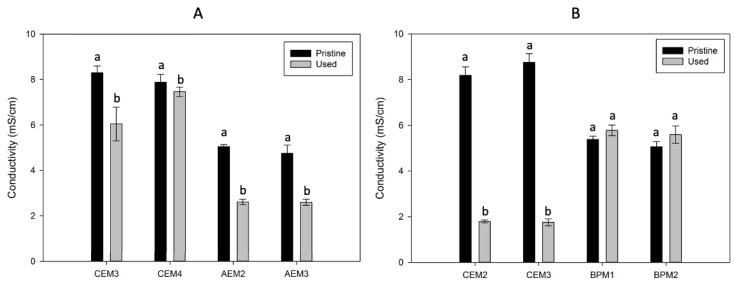
Membrane conductivity before and after the (**A**) ED and (**B**) EDBM treatments. Different letters indicate significant differences between the means (Tukey, *p* < 0.05).

No significant differences were observed in the thickness of the membranes from both the ED and EDBM processes (*p* > 0.05) (data not shown). However, during the ED part of the process, the average conductivity losses were 23% and 16% for CEM3 and CEM4 and 48% and 45% for AEM2 and AEM3 (Figure 3A). As reported in previous works, conductivity losses under 30%, such as for the CEMs, are not considered severe losses [36]. Dufton et al. (2018) and Lemay et al. (2019) also experienced less than a 30% decrease in membrane conductivity after ED treatment of sweet whey [4,27]. They attributed this decrease to an ion replacement inside the membrane. Indeed, counterions present in the initial membranes can be replaced by divalent ionic species of tofu whey with lower conductivity values [34]. However, the AEMs were more affected by the conductivity loss, with more than 30%. Therefore, this could indicate a more important problem, such as fouling or scaling, since these phenomena are known to decrease the conductivity and permselectivity of membranes [13,14]. Regarding the EDBM part, the conductivity of the BPMs did not change after the EDBM treatments (Figure 3B). However, the CEMs were particularly affected by a conductivity loss, with losses of 78% for the CEM2 and 80% for the CEM3 (Figure 3B). This could indicate potential scaling by the divalent ions present in tofu whey. Indeed, during EDBM, the BPM2 generates OH^−^ ions in the recovery compartment, which alkalinizes the solution. In parallel, divalent cations of the tofu solution migrate into the recovery compartment through CEM2. In this alkaline environment on the surface of the membrane, divalent cations from the tofu whey can form hydroxides and precipitate on the adjacent membrane, the CEM2 [37,38]. The CEM3 would also be affected because the alkalinized recovery solution was recirculated in another compartment adjacent to the CEM3, also containing divalent ions that have migrated from tofu whey (Figure 2). This can explain the drop in conductivity of CEM2 and CEM3, since divalent ions are less conductive than monovalent ions and can form scaling on the surface and inside the membrane [39].

### 3.2. Ash and Mineral Contents

**Figure 4:**
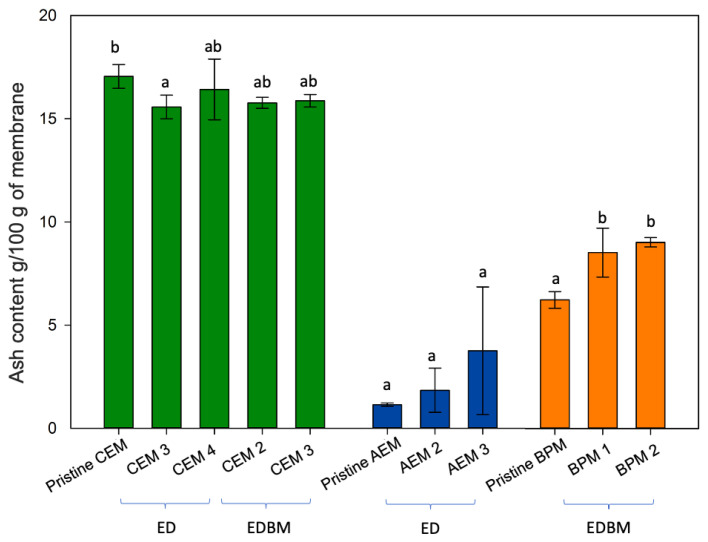
Ash content of the membranes before and after the ED + EDBM process. Different letters indicate significant differences between the pristine membranes and their respective used BP, AEM or CEM (Tukey, *p* < 0.05).

Comparing the ash content of pristine and used membranes can provide information about the presence of scaling. A difference in the ash content may indicate the presence of mineral precipitate or a change in mineral composition. The ash content of the pristine and used CEMs showed no significant difference, except for the CEM3, which presented a small but significant (*p* = 0.023) reduction in the ash content in comparison to the pristine one. However, the ash content of CEM3 was significantly not different than the other used CEMs (Figure 4). Similarly, the ash content of the pristine and used AEMs showed no significant difference. Finally, a slight increase in the ash content was observed in the used BPMs compared to the pristine membrane (*p* < 0.021) (Figure 4).

Following the results of the minerals, some differences were also noticed (Table 3). Regarding the CEMs from both ED and EDBM, the pristine and used ones had similar contents of Na^+^, K^+^ and P. However, higher concentrations of Ca^2+^ and Mg^2+^ were found in the used membranes compared to the pristine one. These ions are contained in tofu whey from the uses of coagulants in the tofu production [40]. Regarding the Ca^2+^ content initially present in the tofu whey, the content found in the used membranes represents only 0.2% of this initial content for CEMs 3 and 4 from the ED process and 0.1% for CEMs 2 and 3 from the EDBM process. For Mg^2+^, the content found in the used membranes represents 0.005% of the initial tofu whey for the CEMs from the ED process and 0.08% for the CEMs from the EDBM process.

Regarding the AEMs, the only difference between the pristine and used ones was in the phosphorus content, which increased for the used AEMs, as also reported by Dufton et al. [4]. In soy products, phosphorus is mainly present in the form of phytic acid, which is negatively charged under a large pH range, including the operating pH values for the ED and EDBM processes [41,42]. Therefore, part of the phytic acid could migrate through the AEMs, explaining the higher content of phosphorus in the used AEMs. In addition, since phytic acid is less conductive than anions, their presence in the AEMs can contribute to the drop in conductivity of the membranes. Surprisingly, despite its high molecular weight (660.04 g/mol), part of the phytic acid could have penetrated the AEMs, probably due to its high density of negative charges [43]. Indeed, in the literature, the penetration into the AEMs of other molecules of similar size has already been reported, such as Quecertin-3-glucoside (464 g/mol) [17]. The content of P in the membrane represents 0.02% of the P content initially present in tofu whey before ED. Finally, for the BPMs, the Na^+^ content was lower in the used membranes, while the Ca^2+^ and Mg^2+^ contents were higher, probably due to interactions with the cation exchange layer. As reported in the literature, during electrodialytic processes, some Na^+^ ions initially present in membranes are replaced by other ions from the treated solution [27,44]. The amounts of Ca^2+^ and Mg^2+^ in the used BPMs represent only 0.2% of the Ca^2+^ and Mg^2+^ initially present in the tofu whey before EDBM.

### 3.3. Ion-Exchange Capacity

The IEC of the CEMs and AEMs showed no significant difference between the pristine and used membranes (*p* = 0.644 and *p* = 0.363) (Table 4). The values were similar to those reported in the literature: 2.18 and 1.56 mmol/g for CMX-fg and AMX-fg, respectively [22]. Although there was no significant difference between the pristine and used membranes, the IEC of the used CEMs and AEMs tended to be lower than that of the pristine membranes. This could indicate a potential decrease in the number of fixed groups, which could be caused by the electrostatic interactions with highly hydrated colloidal structures such as proteins on the surface of the membrane or carboxylic acids, amino acids, and mineral salts on the surface or inside the membrane [45].

### 3.4. Scanning Electron Microscope and Elemental Analysis

**Figure 5:**
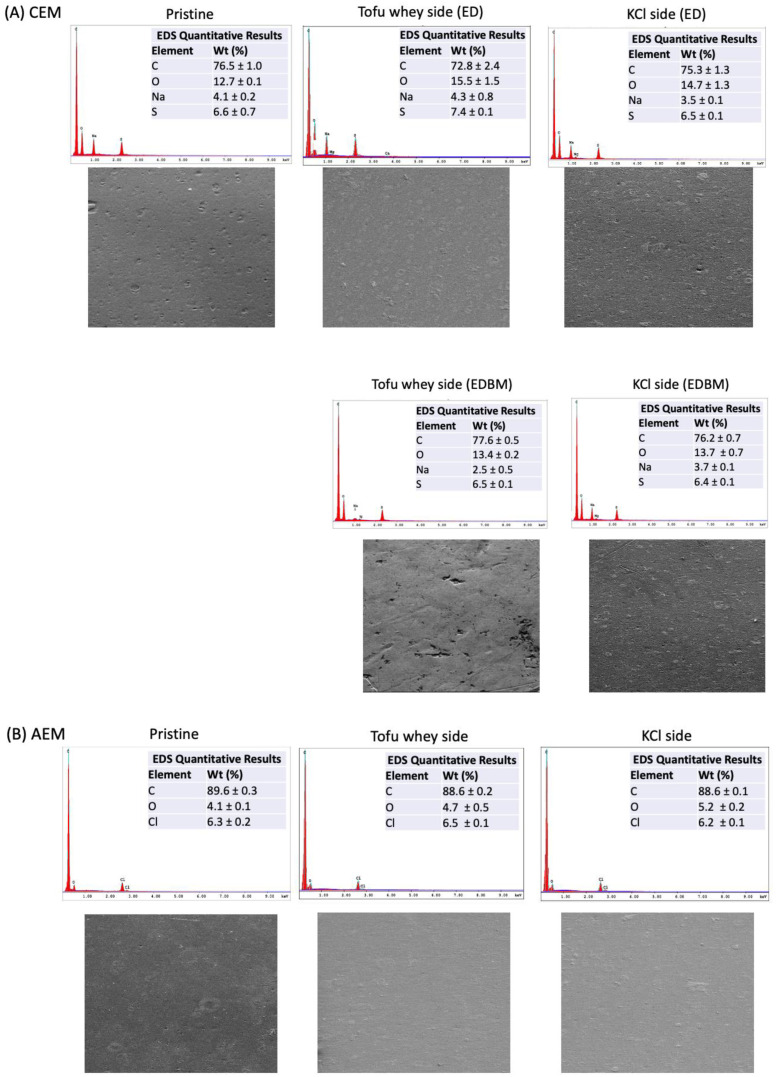
Scanning electron microscopy (SEM) images and elemental analysis (EDX) of the pristine and used (**A**) CEM and (**B**) AEM. The conditions were a 5 kV accelerating voltage with a 9.5–10.5 mm working distance and 400× magnification.

SEM and EDX analyses were performed on the pristine and used membranes to determine potential scaling and identify the minerals responsible for it (Figure 5). No sign of fouling deposits was observed on the surfaces of the pristine and used membranes, whatever the membrane types, AEMs, CEMs or BPMs. In other studies, when scaling by Mg^2+^ or Ca^2+^ occurred on the surface of the membranes, this was clearly demonstrated by the SEM.

Similarly, the elemental analysis did not evidence the presence of mineral deposition on any membranes, since similar element contents were found on the surfaces of the used and pristine membranes. Regarding the CEMs, the elemental analysis detected the presence of carbon, oxygen and sulfur from the polymeric matrix and fixed groups of the membrane, as well as sodium from the counterions used to neutralize the fixed groups [44]. Regarding the AEMs, the elements found were carbon and oxygen from the membrane matrix and chloride, the counter ion used for the neutralization. Similarly to the CEMs, the cationic side of the BPMs contained carbon, oxygen and sodium. Additionally, the anionic side of the BPMs had a similar content to the AEMs, consisting of carbon, oxygen and chloride. No difference was observed between the pristine and used BPMs (data not shown due to a confidential agreement with the membrane manufacturer).

However, SEM-EDX only analyzes the surface of the membrane and does not provide complete information on the presence of minerals inside the membrane. Indeed, SEM analyzes membranes with a penetration depth of approximately 0.5 to 5 µm [46], while the thickness of our membranes varied from approximately 140 to 230 µm depending on the type (AEM, CEM and BPM). Since no change in minerals was observed on the surface of the membranes, it would indicate that the increase in divalent ions reported by the ICP analysis would be localized inside the membrane, probably in transition as they migrate rather than precipitate on the surface [47,48]. Therefore, there is no membrane scaling on these membranes.

### 3.5. FTIR Spectra

**Figure 6:**
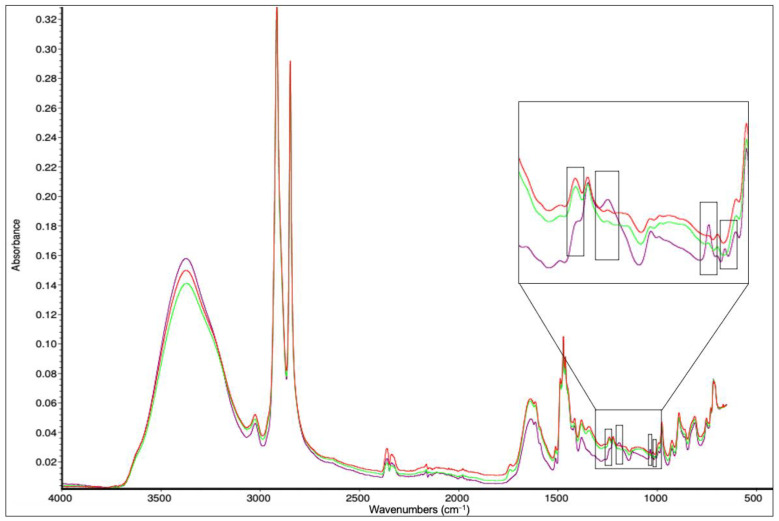
FTIR spectra of the AEMs, pristine membrane in red, tofu whey side in purple and KCl side in green.

No changes were observed for the CEMs’ and BPMs’ absorption spectra before and after the ED + EDBM treatments (data not shown). On the contrary, the FTIR spectra of the AEM and both sides of a used AEM (tofu whey and KCl side) are presented in Figure 6. By comparing them, changes in the spectra of the tofu whey side of the AEM were observed. Indeed, new peaks were observed around 1010 cm^−1^, 1050 cm^−1^ and 1190 cm^−1^, which could be associated with the stretching vibration of the C–OH, C–O bonds and COOH group of phenol [17,49,50]. Therefore, this could indicate the adsorption of a phenolic compound from the tofu whey solution on the membrane interface in contact with the tofu whey. Finally, no peaks were observed for amide I (1630–1635 cm^–1^) and amide II (1517–1526 cm^–1^) [50], indicating the absence of protein fouling. However, the soaking step in NaCl performed for the conductivity analysis may potentially have removed proteins if they were present. But, if this potential protein fouling has been removed by simple soaking in a NaCl solution, this means that this deposition of protein would be reversible and easy to eliminate.

Conversely, some peaks present in the spectra of the pristine AEM and the KCl side of the used AEMs are absent from the tofu whey side of the membranes. Among these, there is the band at 1250 cm^−1^, which may correspond to the C–N stretching vibrations of quaternary ammonium, the functional sites of the membrane [16,51,52]. The absence of this peak could indicate an interaction between the functional sites and a molecule from tofu whey, such as polyphenol. The other absent peaks, at about 1750 cm^−1^ and 1350 cm^−1^, could not be identified.

### 3.6. Isoflavone Concentration

**Figure 7:**
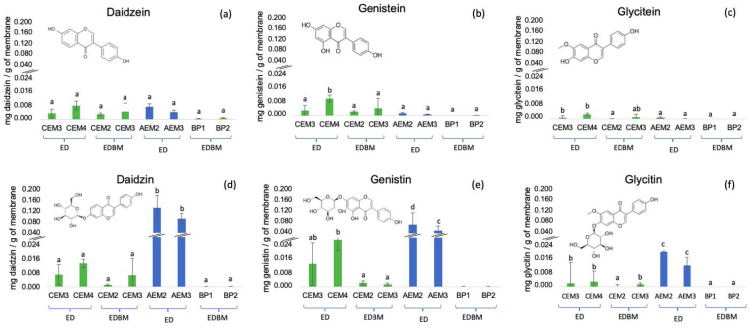
Isoflavones content on the BPMs, AEMs and CEMs after the ED + EDBM process. Different letters indicate significant differences between the pristine membranes and their respective used BP, AEM or CEM (Tukey, *p* < 0.05).

Isoflavones are flavonoid phenolic compounds. Their structure is made of a flavone nucleus, composed of two benzene rings linked to a heterocyclic ring. The main isoflavones in soybean are the aglycone genistein, daidzein, glycitein and their respective glycosylated forms [53]. Figure 7 shows the concentration of aglycone (a, b, c) and glycosylated (d, e, f) isoflavones (mg of isoflavone/g of dry membrane) on the ED and EDBM membranes. The glycosylated forms of isoflavones (glycitin, daidzin and genistin) (Figure 7d–f) were present in more important concentrations on these membranes compared to their respective aglycone forms (glycitein, daidzein and genistein). In addition, a higher concentration of glycosylated forms was found on the AEMs, while small amounts were found on the CEMs and only traces on the BPMs. Moreover, the CEMs from the ED process contain a higher concentration of isoflavones than the CEMs from the EDBM process.

Regarding the concentration of the different isoflavones, some differences were noticed. On the AEMs, daidzein was present in a concentration about 2.5× higher than genistein and 7× higher than glycitein, while on the CEMs, daidzin and genistin were present in a similar concentration, about 4X higher than glycitin.

**Figure 8:**
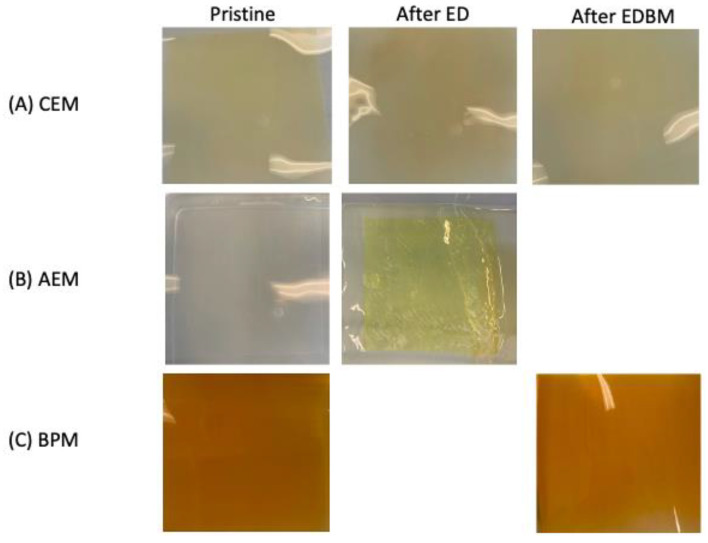
Photographs of the pristine and used (**A**) CEM, (**B**) AEM and (**C**) BPM.

Photographs of the membranes are presented in Figure 8. Visually, there was no difference in the appearance and texture of the CEMs and BPMs (Figure 8A,C), which provides no evidence of fouling. Indeed, as shown previously in many works, when fouling or scaling occurs, mineral or protein deposits are visually apparent [4,18]. However, the AEMs presented a yellow coloration after the ED treatments (Figure 8B). The yellow coloration could be due to the migration of isoflavone. Indeed, soy isoflavones, such as genistein and daidzein, are yellow-pigmented [54]. No difference was observed in the textures of the membranes.

## 4. Discussion

### 4.1. Scaling

Actually, it is not possible to affirm that there is no scaling on the membranes (CEMs, AEMs and BPMs). Despite the ICP-OES analysis highlighting a higher concentration of divalent ions in the CEMs and BPMs, these ions seem to be in transit in the membranes rather than forming a scaling. Regarding the AEMs, no scaling was observed and the change in membrane conductivity would be due to the higher phosphorus ion content. Such an increase in the phosphorus ion content would be caused by phytic acid in transit through the membrane. Since the organic acids are less mobile than the anions, the conductivity of the membrane was reduced. In addition, following the procedure of Casademont et al. (2008) to ensure the presence or otherwise of a fouling [26], after three consecutive ED and EDBM processes, the respective total durations were 429 and 102 min. However, although no scaling was observed, we cannot fully exclude, for longer durations more representative of the industrial conditions, the possibility of an accumulation of divalent ions in the membranes, potentially leading to internal scaling or membrane poisoning [55]. Similar analyses would be necessary to evaluate the scaling in the longer term.

### 4.2. Fouling

To the best of our knowledge, no work has been carried out on isoflavone fouling or reported membrane fouling by isoflavones. However, there are many articles in the literature on the fouling of CEMs and AEMs by other polyphenols, such as anthocyanins, PACs and phenolic acids [12,16,17,23,49,56,57]. These studies established that the possible interactions between the IEMs (CEM and AEM) and these polyphenols are electrostatic interactions between fixed groups of the membrane and polyphenolic ions, hydrophobic–hydrophobic π–π stacking interactions between aromatic rings of polyphenols and the IEM material containing aromatic rings, and hydrogen bonds between hydroxyl or carboxyl groups of polyphenols and oxygen of fixed groups or hydrogen of the aliphatic chains of membranes [17,23,58]. For BPMs, no studies reported interactions between polyphenols and the BPMs. However, since BPMs are formed of a cationic and anionic layer, the same could apply, but in the present study, it was demonstrated that polyphenols did not interact with the BPMs.

**Figure 9:**
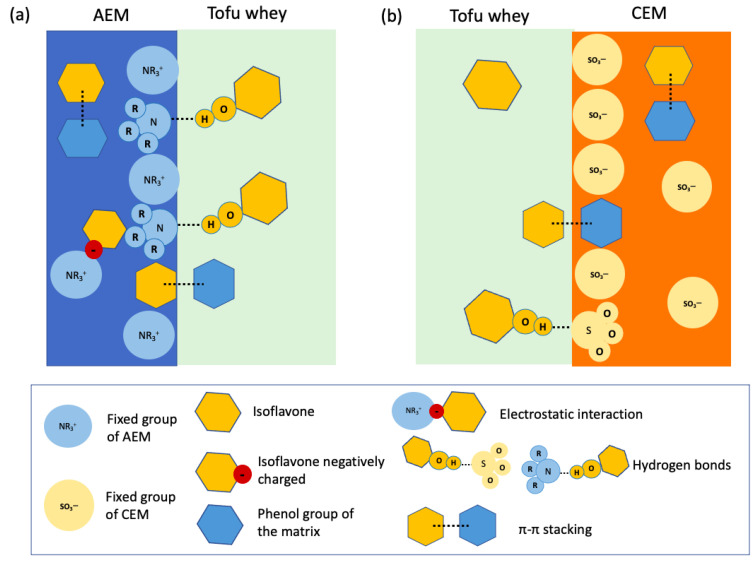
Schematic representation of the possible interaction between isoflavones and (**a**) AEM and (**b**) CEM.

AMX-fg and CMX-fg are homogeneous membranes made of an aromatic polystyrene and divinylbenzene matrix, with their respective functional groups, NR^3+^ and SO_3_^−^, chemically bonded, which makes a single phase extended throughout the entire membrane [59]. Hence, regarding the structure of our membranes, the adsorption of isoflavones on the membranes may be due, for the AEMs, to a combination of these three intermolecular interactions (electrostatic interactions, hydrogen bond and π–π stacking) (Figure 9a) and for the CEMs, only to π–π stacking and hydrogen bonds (Figure 9b).

For the AEMs, concerning the electrostatic interactions, despite the IEC of the membranes showing no significant differences, the slight reduction may be caused by some electrostatic interactions between the isoflavones and fixed groups of the AEMs. Indeed, electrostatic interactions rely on the charge of both the isoflavones and fixed groups of membranes. Regarding isoflavones, their electrical charge depends on the pH of the solution, causing protonation and deprotonation of hydroxyl groups. The pKa1 and pKa2 values of daidzein are 7.51 ± 0.07 and 9.47 ± 0.14, and those of genistein are 7.25 ± 0.84 and 9.53 ± 0.15 [60]. The pKa values of other isoflavones are not reported in the literature. At the pH of the tofu whey solution during the ED and EDBM processes, which is between 6.11 and 4.4, isoflavones are mostly non-charged, with 96.2–99.9% of daidzein and 99.3–99.86% of genistein not deprotonated, calculated according to the acid dissociation equation [61]. Therefore, electrostatic interactions between phenol and AEM were unlikely [62]. However, due to the Donnan exclusion, the membrane’s internal pH can be different from the external solution’s pH. In the case of the AEMs, the internal pH can be more alkaline by 2–3 pH units [58]. At these pH values, part of the isoflavones can be negatively charged because of the deprotonation of hydroxyl groups since the pH is higher than the pKa1 of isoflavones. Indeed, for an increase of 3 pH units, 43.7–97.58% of daidzein and 58.6–98.6% of genistein would be deprotonated according to the calculation by the acid dissociation equation. Thus, some electrostatic interactions can possibly occur between the negatively charged isoflavones and NR^3+^ groups fixed on the AEMs [16,17]. In addition, the aglycone forms of isoflavones have fewer OH^−^ groups [63] and, therefore, are less prone to ionize and interact with membranes via electrostatic interactions, which could explain their lower concentrations on the AEMs. In addition to electrostatic interactions, hydrogen bonds are possible between the secondary or tertiary amine groups of the AEMs’ fixed groups and the hydroxyl groups of phenol [58] (Figure 9a). The higher content of OH^−^ groups in the glycosylated form of isoflavones could allow for higher amounts of hydrogen bonds with the membrane, which could explain their higher concentration on the AEMs compared to the aglycone forms. Finally, π–π stacking, a noncovalent interaction involving aromatic groups containing π bonds [64], can occur, as evidenced in other studies regarding the adsorption of other polyphenols on ion-exchange membranes. These interactions occur between the benzene rings present in the polystyrene and divnylbenzene from the matrix of the membranes and phenol rings of the isoflavone [19,62,65].

For the CEMs, since they are negatively charged because of the SO_3_^−^ groups, electrostatic interactions with isoflavones are unlikely. In addition, as mentioned earlier, the IEC of the membranes showed no significant differences. Therefore, only π–π stacking interactions between the membrane’s matrix of CEMs and isoflavones and hydrogen bonds with fixed groups can occur (Figure 9b) [17,19,62,65].

As reported by Pismenskaya et al. (2020) for anthocyanins, isoelectric interactions are the predominant ones and allow higher adsorption of these molecules on anion-exchange resins compared to hydrogen bond and π–π stacking interactions. Indeed, when no electrostatic interactions were formed because of the pH of the solution, the concentration of anthocyanins on the membranes was lower [65]. This goes the same way as our results. Indeed, for the AEMs, the three interactions are possible (electrostatic interactions, hydrogen bonds and π–π stacking), while only π–π stacking and hydrogen bonds are possible for the CEMs, where the isoflavone concentration was found to be lower [17,19,62,65].

In addition, the higher concentration of isoflavones in the CEMs from the ED compared to the EDBM process may be caused by the different durations of the processes. Indeed, the ED lasted much longer than the EDBM (143 min compared to 34 min). Therefore, the longer duration of contact of the isoflavones on the ED membranes can explain the higher concentration, as also reported by Bdiri et al. (2020) on anthocyanins [17].

Finally, the difference in the concentration of isoflavones on the membranes can be explained by their relative abundance in tofu whey, as well as the number of functional groups that can interact with the membranes. Regarding the functional groups, genistin has three hydroxyl groups, while daidzin has two and glycitin has two, in addition to a methoxy group. However, daidzin was present in higher concentrations, particularly in the AEMs. The higher concentration of daidzin could, therefore, be explained by their abundance in tofu whey. Indeed, generally, genistin, daidzin, and glycitin represent approximately 50%, 40%, and 10% of soy isoflavones, and the aglycone forms are present in higher concentrations. These proportions vary by many factors, such as the soy variety and processing conditions. [66,67]. In tofu whey, daidzein and genistein are present in the highest concentrations [68].

## 5. Conclusions

In this study, the fouling of the different membranes at each step of an ED + EDBM process for protein recovery from tofu whey was investigated. The analyses did not evidence any scaling by divalent ions present in the tofu whey after three runs of the process (429 min. for and 102 min. for EDBM) with the same sets of membranes. The differences observed in the mineral composition of the membranes would instead be due to the transfer of ions during the process, which could explain the drop observed in the conductivity in the CEMs and AEMs. On another note, for the first time, interactions between isoflavones and IEMs were reported. Indeed, the AEMs were particularly affected and presented a yellow coloration due to the adsorption of isoflavones. Moreover, the glycosylated forms of the isoflavones were present on the membranes in higher concentrations than the aglycone forms, probably due to the higher content of hydroxyl groups, allowing more hydrogen interactions with the membranes. Regarding the CEMs, the possible interactions with isoflavone were via π–π stacking between phenol groups of the isoflavone and the membrane’s matrix and hydrogen bonds with fixed groups. The higher concentration of isoflavones on the AEMs could be explained by the combination of electrostatic interactions, hydrogen bonding, and π–π stacking due to the charge of the fixed groups and the nature of the polymer matrix.

## Figures and Tables

**Table 1:** Initial and concentrated tofu whey proximal composition (data from Deschênes-Gagnon, et al. [3]).

Component	Unit	Initial Tofu Whey	3× Tofu Whey
Protein	g/100 g liquid basis	0.42 ± 0.01 ^a^	1.18 ± 0.01 ^b^
Ash		0.51 ± 0.01 ^a^	0.81 ± 0.01 ^c^
Ca^2+^		0.02 ± 0.01 ^a^	0.05 ± 0.01 ^b^
K^+^		0.14 ± 0.01 ^a^	0.21 ± 0.02 ^b^
Mg^2+^		0.03 ± 0.01 ^a^	0.08 ± 0.01 ^b^
Na^+^		0.03 ± 0.01 ^a^	0.04 ± 0.01 ^a^
P		0.01 ± 0.01 ^a^	0.02 ± 0.01 ^b^
Stachyose		0.40 ± 0.01 ^a^	1.16 ± 0.04 ^b^
Raffinose		0.13 ± 0.01 ^a^	0.27 ± 0.08 ^b^
Sucrose		0.28 ± 0.01 ^a^	0.69 ± 0.03 ^b^
Lipid		0.10 ± 0.01	
Dry matter		2.79	7.03
pH		6.1 ± 0.1 ^a^	6.1 ± 0.1 ^a^
Conductivity	mS/cm	5.9 ± 0.4 ^a^	7.0 ± 0.2 ^b^

Different letters on the same line indicate significant differences between the means (Tukey’s test, *p* < 0.05).

**Table 2:** Structural characteristics of the membranes.

		Fixed Groups	Matrix	Type	Ion Exchange Capacity (mmol/g)	Thickness (mm)
AMX-Fg [20,21,22]		NR^3+^	Polystyrene and Divinylbenzene (PS + DVB)	Homogeneous	1.5–1.8	120–180
CMX-Fg [21,22,23,24]		SO_3_^−^	Polystyrene and Divinylbenzene (PS + DVB)	Homogeneous	1.4–1.7	140–200
BP-1E [25]	Anion-exchange layer	NR^3+^	Polysulfone	Homogeneous	-	0.22
	Cation-exchange layer	SO_3_^−^	Reinforced Neosepta CM-1			

**Table 3:** Mineral characterization of the membranes before and after the ED + EDBM treatments.

	mg/g of Membrane		Na	K	P	Ca	Mg
ED	CEM	Pristine	24.2 ± 1.3 ^a^	1.9 ± 0.7 ^a^	0.03 ± 0.03 ^a^	0.7 ± 0.2 ^a^	0.0 ± 0.0 ^a^
		CEM 3	22.5 ± 1.2 ^a^	1.9 ± 2.1 ^a^	0.06 ± 0.03 ^a^	1.9 ± 0.2 ^b^	0.6 ± 0.6 ^b^
		CEM 4	25.2 ± 1.3 ^a^	0.9 ± 0.3 ^a^	0.07 ± 0.09 ^a^	1.7 ± 0.1 ^b^	0.7 ± 0.6 ^b^
	AEM	Pristine	0.4 ± 0.1 ^a^	0.2 ± 0.2 ^a^	0.01± 0.00 ^a^	0.1 ± 0.1 ^a^	0.0 ± 0.0 ^a^
		AEM 2	0.7 ± 0.2 ^a^	0.1 ± 0.1 ^a^	0.08 ± 0.02 ^b^	0.1 ± 0.1 ^a^	0.0 ± 0.0 ^a^
		AEM 3	0.4 ± 0.4 ^a^	0.1 ± 0.1 ^a^	0.09 ± 0.04 ^b^	0.1 ± 0.1 ^a^	0.0 ± 0.0 ^a^
EDBM	CEM	CEM 2	19.8 ± 2.4 ^a^	1.5 ± 0.6 ^a^	0.02 ± 0.01 ^a^	1.4 ± 0.1 ^c^	1.9 ± 0.3 ^b^
		CEM 3	14.4 ± 3.5 ^a^	1.4 ± 0.7 ^a^	0.03 ± 0.03 ^a^	1.2 ± 0.2 ^b^	1.8 ± 0.1 ^b^
	BPM	Pristine	18.8 ± 0.7 ^b^	2.6 ± 1.2 ^a^	0.02 ± 0.02 ^a^	0.2 ± 0.0 ^a^	0.0 ± 0.0 ^a^
		BPM 1	10.7 ± 2.5 ^a^	1.8 ± 0.2 ^a^	0.13 ± 0.18 ^a^	1.0 ± 0.1 ^b^	1.2 ± 0.2 ^b^
		BPM 2	12.8 ± 2.0 ^a^	2.4 ± 0.8 ^a^	0.16 ± 0.24 ^a^	1.4 ± 0.1 ^c^	1.8 ± 0.2 ^c^

Different letters indicate significant differences between the pristine membranes and their respective used BP, AEM or CEM (Tukey, *p* < 0.05).

**Table 4:** Average values of the ion-exchange capacity (IEC) of the pristine and used AEMs and CEMs.

	(meq/g)	Pristine	Used
ED	CEM	2.6 ± 0.6 ^a^	2.0 ± 0.7 ^a^
	AEM	1.9 ± 0.1 ^a^	1.6 ± 0.2 ^a^
EDBM	CEM	2.6 ± 0.6 ^a^	2.5 ± 0.7 ^a^

Different letters indicate significant differences between the pristine membranes and their respective used BP, AEM or CEM (Tukey, *p* < 0.05).

## Data Availability

The data presented in this study are available on request from the corresponding author. The data are not publicly available due to ongoing industrial collaborative projects.

## References

[1] Yu S.S., Ahn H.S., Park S.H. (2023). Potential application of electrical conductivity measurement for freshness assessment of tofu. Sens. Actuators A Phys..

[2] Bazinet L., Ippersiel D., Lamarche F. (1999). Recovery of magnesium and protein from soy tofu whey by electrodialytic configurations. J. Chem. Technol. Biotechnol..

[3] Deschênes-Gagnon R., Langevin M., Lutin F., Bazinet L. (2023). Impact of nanofiltration pre-concentration prior to a combination of electrodialytic processes on the extraction of proteins from tofu whey. Future Foods.

[4] Dufton G., Mikhaylin S., Gaaloul S., Bazinet L. (2018). How electrodialysis configuration influences acid whey deacidification and membrane scaling. J. Dairy Sci..

[5] Gazigil L., Er E., Kestioğlu O.E., Yonar T. (2022). Pilot-Scale Test Results of Electrodialysis Bipolar Membrane for Reverse-Osmosis Concentrate Recovery. Membranes.

[6] Spettmann D., Eppmann S., Flemming H.-C., Wingender J. (2007). Simultaneous visualisation of biofouling, organic and inorganic particle fouling on separation membranes. Water Sci. Technol..

[7] Sadr S.M.K., Saroj D.P., Basile A., Cassano A., Rastogi N.K. (2015). 14-Membrane technologies for municipal wastewater treatment. Advances in Membrane Technologies for Water Treatment.

[8] Pal P. (2020). Chapter 1—Introduction to membrane materials, processes, and modules. Membrane-Based Technologies for Environmental Pollution Control.

[9] Guo H., Kim Y. (2021). Membrane Scaling in Electrodialysis Fed with High-Strength Wastewater. Environ. Eng. Sci..

[10] Lee H.-J., Hong M.-K., Han S.-D., Cho S.-H., Moon S.-H. (2009). Fouling of an anion exchange membrane in the electrodialysis desalination process in the presence of organic foulants. Desalination.

[11] Bdiri M., Bensghaier A., Chaabane L., Kozmai A., Baklouti L., Larchet C. (2019). Preliminary Study on Enzymatic-Based Cleaning of Cation-Exchange Membranes Used in Electrodialysis System in Red Wine Production. Membranes.

[12] Tsygurina K., Pasechnaya E., Chuprynina D., Melkonyan K., Rusinova T., Nikonenko V., Pismenskaya N. (2022). Electrodialysis Tartrate Stabilization of Wine Materials: Fouling and a New Approach to the Cleaning of Aliphatic Anion-Exchange Membranes. Membranes.

[13] Thompson D., Tremblay A. (1983). Fouling in steady and unsteady state electrodialysis. Desalination.

[14] Bleha M., Tishchenko G., Šumberová V., Kůdela V. (1992). Characteristic of the critical state of membranes in ED-desalination of milk whey. Desalination.

[15] Dammak L., Pismenskaya N. (2021). In-Depth on the Fouling and Antifouling of Ion-Exchange Membranes. Membranes.

[16] Bdiri M., Dammak L., Larchet C., Hellal F., Porozhnyy M., Nevakshenova E., Pismenskaya N., Nikonenko V. (2019). Characterization and cleaning of anion-exchange membranes used in electrodialysis of polyphenol-containing food industry solutions; comparison with cation-exchange membranes. Sep. Purif. Technol..

[17] Bdiri M., Perreault V., Mikhaylin S., Larchet C., Hellal F., Bazinet L., Dammak L. (2020). Identification of phenolic compounds and their fouling mechanisms in ion-exchange membranes used at an industrial scale for wine tartaric stabilization by electrodialysis. Sep. Purif. Technol..

[18] Mikhaylin S., Sion A.-V. (2016). Improvement of a sustainable hybrid technology for caseins isoelectric precipitation (electrodialysis with bipolar membrane/ultrafiltration) by mitigation of scaling on cation-exchange membrane. Innov. Food Sci. Emerg. Technol..

[19] Ge S., Zhang Z., Yan H., Irfan M., Xu Y., Li W., Wang H., Wang Y. (2020). Electrodialytic Desalination of Tobacco Sheet Extract: Membrane Fouling Mechanism and Mitigation Strategies. Membranes.

[20] Pismenskaya N., Rybalkina O., Solonchenko K., Pasechnaya E., Sarapulova V., Wang Y., Jiang C., Xu T., Nikonenko V. (2023). How Chemical Nature of Fixed Groups of Anion-Exchange Membranes Affects the Performance of Electrodialysis of Phosphate-Containing Solutions?. Polymers.

[21] Sarapulova V., Shkorkina I., Mareev S., Pismenskaya N., Kononenko N., Larchet C., Dammak L., Nikonenko V. (2019). Transport Characteristics of Fujifilm Ion-Exchange Membranes as Compared to Homogeneous Membranes AMX and CMX and to Heterogeneous Membranes MK-40 and MA-41. Membranes.

[22] Ozkul S., van Daal J.J., Kuipers N.J., Bisselink R.J., Bruning H., Dykstra J.E., Rijnaarts H.H. (2023). Transport mechanisms in electrodialysis: The effect on selective ion transport in multi-ionic solutions. J. Membr. Sci..

[23] Perreault V., Sarapulova V., Tsygurina K., Pismenskaya N., Bazinet L. (2021). Understanding of Adsorption and Desorption Mechanisms of Anthocyanins and Proanthocyanidins on Heterogeneous and Homogeneous Cation-Exchange Membranes. Membranes.

[24] Luo T., Roghmans F., Wessling M. (2020). Ion mobility and partition determine the counter-ion selectivity of ion exchange membranes. J. Membr. Sci..

[25] Pärnamäe R., Mareev S., Nikonenko V., Melnikov S., Sheldeshov N., Zabolotskii V., Hamelers H.V.M., Tedesco M. (2021). Bipolar membranes: A review on principles, latest developments, and applications. J. Membr. Sci..

[26] Casademont C., Pourcelly G., Bazinet L. (2008). Effect of magnesium/calcium ratios in solutions treated by electrodialysis: Morphological characterization and identification of anion-exchange membrane fouling. J. Colloid Interface Sci..

[27] Lemay N., Mikhaylin S., Bazinet L. (2019). Voltage spike and electroconvective vortices generation during electrodialysis under pulsed electric field: Impact on demineralization process efficiency and energy consumption. Innov. Food Sci. Emerg. Technol..

[28] Lteif R., Dammak L., Larchet C., Auclair B. (1999). Conductivitéélectrique membranaire: Étude de l’effet de la concentration, de la nature de l’électrolyte et de la structure membranaire. Eur. Polym. J..

[29] Lebrun L., Da Silva E., Pourcelly G., Métayer M. (2003). Elaboration and characterisation of ion-exchange films used in the fabrication of bipolar membranes. J. Membr. Sci..

[30] AOAC (1995). Method 945.46: Ash in Milk. Official Methods of Analysis of AOAC International.

[31] Ayala-Bribiesca E., Boucher M., Bazinet L. (2012). Ultrathin Sicopion Composite Cation-Exchange Membranes: Characteristics and Electrodialytic Performance following a Conditioning Procedure. Int. J. Chem. Eng..

[32] Sneddon J., Vincent M.D. (2008). ICP-OES and ICP-MS for the Determination of Metals: Application to Oysters. Anal. Lett..

[33] Guo H., You F., Yu S., Li L., Zhao D. (2015). Mechanisms of chemical cleaning of ion exchange membranes: A case study of plant-scale electrodialysis for oily wastewater treatment. J. Membr. Sci..

[34] Ayala-Bribiesca E., Pourcelly G., Bazinet L. (2006). Nature identification and morphology characterization of cation-exchange membrane fouling during conventional electrodialysis. J. Colloid Interface Sci..

[35] Zhang S., Zheng Z.-P., Zeng M.-M., He Z.-Y., Tao G.-J., Qin F., Chen J. (2017). A novel isoflavone profiling method based on UPLC-PDA-ESI-MS. Food Chem..

[36] Kravtsov V., Kulikova I., Mikhaylin S., Bazinet L. (2019). Alkalinization of acid whey by means of electrodialysis with bipolar membranes and analysis of induced membrane fouling. J. Food Eng..

[37] Gence N., Ozbay N. (2006). pH dependence of electrokinetic behavior of dolomite and magnesite in aqueous electrolyte solutions. Appl. Surf. Sci..

[38] Mikhaylin S., Nikonenko V., Pourcelly G., Bazinet L. (2016). Hybrid bipolar membrane electrodialysis/ultrafiltration technology assisted by a pulsed electric field for casein production. Green Chem..

[39] Speight J. (2005). Lange’s Handbook of Chemistry.

[40] Chua J.-Y., Liu S.-Q. (2019). Soy whey: More than just wastewater from tofu and soy protein isolate industry. Trends Food Sci. Technol..

[41] Costello A.J., Glonek T., Myers T.C. (1976). 31P Nuclear magnetic resonance pH titrations of myo-inositol hexaphosphate. Carbohydr. Res..

[42] Cheryan M., Rackis J.J. (1980). Phytic acid interactions in food systems. C R C Crit. Rev. Food Sci. Nutr..

[43] Nassar M., Nassar R., Maki H., Al-Yagoob A., Hachim M., Senok A., Williams D., Hiraishi N. (2021). Phytic Acid: Properties and Potential Applications in Dentistry. Front. Mater..

[44] Veerman J., Vermaas D.A., Cipollina A., Micale G. (2016). 4—Reverse electrodialysis: Fundamentals. Sustainable Energy from Salinity Gradients.

[45] Pismenskaya N., Bdiri M., Sarapulova V., Kozmai A., Fouilloux J., Baklouti L., Larchet C., Renard E., Dammak L. (2021). A Review on Ion-Exchange Membranes Fouling during Electrodialysis Process in Food Industry, Part 2: Influence on Transport Properties and Electrochemical Characteristics, Cleaning and Its Consequences. Membranes.

[46] Marie E., Torbjörn W., Johansson I., Somasundaran P. (2007). 4—Surface Analytical Techniques Applied to Cleaning Processes. Handbook for Cleaning/Decontamination of Surfaces.

[47] Firdaous L., Malériat J., Schlumpf J., Quéméneur F. (2007). Transfer of Monovalent and Divalent Cations in Salt Solutions by Electrodialysis. Sep. Sci. Technol..

[48] Dufton G., Mikhaylin S., Gaaloul S., Bazinet L. (2019). Positive Impact of Pulsed Electric Field on Lactic Acid Removal, Demineralization and Membrane Scaling during Acid Whey Electrodialysis. Int. J. Mol. Sci..

[49] Pasechnaya E., Tsygurina K., Ponomar M., Chuprynina D., Nikonenko V., Pismenskaya N. (2023). Comparison of the Electrodialysis Performance in Tartrate Stabilization of a Red Wine Using Aliphatic and Aromatic Commercial and Modified Ion-Exchange Membranes. Membranes.

[50] Nandiyanto A.B.D., Oktiani R., Ragadhita R. (2019). How to Read and Interpret FTIR Spectroscope of Organic Material. Indones. J. Sci. Technol..

[51] Garcia-Vasquez W., Dammak L., Larchet C., Nikonenko V., Pismenskaya N., Grande D. (2013). Evolution of anion-exchange membrane properties in a full scale electrodialysis stack. J. Membr. Sci..

[52] Luo Q., Zhang H., Chen J., Qian P., Zhai Y. (2008). Modification of Nafion membrane using interfacial polymerization for vanadium redox flow battery applications. J. Membr. Sci..

[53] Rostagno M., Manchón N., Guillamón E., García-Lafuente A., Garicochea A., Alfredo M. (2009). Methods and Techniques for the Analysis of Isoflavones in Foods. Chromatography Types, Techniques and Methods.

[54] Kim I.-S. (2021). Current Perspectives on the Beneficial Effects of Soybean Isoflavones and Their Metabolites for Humans. Antioxidants.

[55] Schalenbach M., Keller L., Janotta B., Bauer A., Tempel H., Kungl H., Bonnet M., Eichel R.-A. (2022). The Effect of Ion Exchange Poisoning on the Ion Transport and Conduction in Polymer Electrolyte Membranes (PEMs) for Water Electrolysis. J. Electrochem. Soc..

[56] Bdiri M., Dammak L., Chaabane L., Larchet C., Hellal F., Nikonenko V., Pismenskaya N. (2018). Cleaning of cation-exchange membranes used in electrodialysis for food industry by chemical solutions. Sep. Purif. Technol..

[57] Sarapulova V.V., Klevtsova A.V., Pismenskaya N.D. (2020). Electrostatic Interactions of Ion-Exchange Materials with Anthocyanins in the Processes of Their Sorption and Electrodialysis Extraction from Liquid Media. Membr. Membr. Technol..

[58] Sarapulova V., Nevakshenova E., Nebavskaya X., Kozmai A., Aleshkina D., Pourcelly G., Nikonenko V., Pismenskaya N. (2018). Characterization of bulk and surface properties of anion-exchange membranes in initial stages of fouling by red wine. J. Membr. Sci..

[59] Xu T. (2005). Ion exchange membranes: State of their development and perspective. J. Membr. Sci..

[60] Nan G., Shi J., Huang Y., Sun J., Lv J., Yang G., Li Y. (2014). Dissociation Constants and Solubilities of Daidzein and Genistein in Different Solvents. J. Chem. Eng. Data.

[61] Alongi K.S., Shields G.C. (2010). Theoretical calculations of acid dissociation constants: A review article. Annu. Rep. Comput. Chem..

[62] Ghafari M., Cui Y., Alali A., Atkinson J.D. (2019). Phenol adsorption and desorption with physically and chemically tailored porous polymers: Mechanistic variability associated with hyper-cross-linking and amination. J. Hazard. Mater..

[63] Adandé S. (2006). Séparation des Isoflavones de Soja par Électrodialyse.

[64] Zhuang W.-R., Wang Y., Cui P.-F., Xing L., Lee J., Kim D., Jiang H.-L., Oh Y.-K. (2019). Applications of π-π stacking interactions in the design of drug-delivery systems. J. Control. Release.

[65] Pismenskaya N., Sarapulova V., Klevtsova A., Mikhaylin S., Bazinet L. (2020). Adsorption of Anthocyanins by Cation and Anion Exchange Resins with Aromatic and Aliphatic Polymer Matrices. Int. J. Mol. Sci..

[66] Soyata A., Hasanah A.N., Rusdiana T. (2021). Isoflavones in Soybean as a Daily Nutrient: The Mechanisms of Action and How They Alter the Pharmacokinetics of Drugs. Turk. J. Pharm. Sci..

[67] Zhao C.-C., Lu J.-K., Ameer K. (2021). Effects of tofu whey powder on the quality attributes, isoflavones composition and antioxidant activity of wheat flour pan bread. LWT.

[68] Zhu Y., Wang Z., Zhang L. (2019). Optimization of lactic acid fermentation conditions for fermented tofu whey beverage with high-isoflavone aglycones. LWT.

